# Items

**Published:** 1904-06

**Authors:** 


### ITEMS.

### STATE CIVIL SERVICE EXAMINATION.

*Examination for Superintendent, Rome State Custodial Asylum.*

An open competitive examination will be held at Albany, Buffalo, New York and Syracuse, June 3, 1904, for the position of superintendent of the Rome State Custodial Asylum. The authorised maximum salary is $4,000 with maintenance for the superintendent and family. The statute requires that the superintendent "shall be a resident of this state, a well-educated physician and graduate of a legally incorporated medical college," and must "have had a suitable experience and training of not less than three years in the care and treatment of the mentally defective classes, epileptic or insane."

Subjects of examination and relative weights are, laws relating to the institution and the commitment, care and treatment of the dependent and defective classes, 1; institutional care, training and treatment of the custodial class of feeble-minded persons and idiots, 3; institution supervision, administration and discipline, 2; education, experience and personal qualifications, 4.

Persons desiring to enter the examination must execute applications on forms supplied by the commission and file them in its office before noon of June 1. For application blank, address Chief Examiner, State Civil Service Commission, Albany, N. Y.

---

W. B. SAUNDERS & Co., medical publishers, Philadelphia, invite special attention to a new work, "The vermiform appendix and its diseases," by Howard A. Kelly, M.D., of the Johns Hopkins University, Baltimore, Md. This, they say, will be one of the most magnificent medical books ever published, containing over four hundred superb illustrations, in the preparation of which the artists of the Johns Hopkins University have spent many years.

Another book to which they also call special attention is Dr. Warren Stone Bickham's "Operative surgery," which met with such remarkable success that the large first edition was exhausted in six months. The second edition is just off the press, but as the only changes are the correction of some few typographic errors, it will not be sent out for review.

The JOURNAL presented a comprehensive review of Dr. Bickham's book in its issue for January, 1904, page 419.

